# Mr. Percy Raiment and Mr. Gordon R. Ward

**Published:** 1913-10-18

**Authors:** Gordon R. Ward

**Affiliations:** 2 Perham Crescent, West Kensington, W.


### Mr. Percy Raiment and Mr. Gordon R. Ward.

Sir,—In your last issue occur the following words:—"There was a time when Dr. Ward professed to be an admirer of those Acts (*i.e.*, the Trade Union Acts) which give us these immunities." This remark is made by Dr. Percy Raiment, general secretary of the National Medical Guild, and, in conjunction with a reference to myself as "late assistant secretary to this Guild," are calculated to give a false impression both as to my present views and as to my reason for severing my connection with the Guild.

My present views are those which I have enunciated at various meetings and in your columns; they have not altered. It is true that I have written to the *British Medical Journal* urging a certain course on the Association which is not without danger to any body which lacks the advantage of registration under the Trade Union Acts; but this I have done because the Guild does not appear to be in a position to undertake even that part of the work in connection with "undesirables" which the Association could safely undertake. When the Guild can find someone to devote the necessary time to this work I shall cease to urge its adoption on any other body.

My reasons for leaving the Guild were simple and sufficient. I had persuaded many gentlemen to join on the understanding that the Guild was not intended to be hostile to the B.M.A. At the end of July Dr. Raiment apprised me by letter that he intended to repudiate that attitude. If he wishes the letter published I can forward a copy. I therefore sent in my resignation. This was formally acknowledged, but has not yet, apparently, come before the Council, for I have heard nothing more.

If Dr. Raiment wishes to be free to oppose the B.M.A. he should, to my mind, resign his official positions in connection therewith. That he has not done so supplies me with a reason for doubting the propriety of belonging to any body of which he is the chief executive official.

Finally, in view of the fact that my resignation has not been accepted by the Council of the National Medical Guild, the body which appointed me organising secretary, and alone has power to terminate the appointment, may I ask why I have not received The Hospital for many weeks past?—I am, Sir, yours faithfully,

Gordon R. Ward.

2 Perham Crescent, West Kensington, W.
October 8, 1913.

[We have no knowledge as to why Mr. Gordon Ward received or has discontinued to receive a copy of The Hospital.—Ed. The Hospital.]

